# Dose escalation of Stereotactic Body Radiotherapy (SBRT) for locally advanced unresectable pancreatic cancer patients with CyberKnife: protocol of a phase I study

**DOI:** 10.1186/s13014-016-0760-1

**Published:** 2017-01-09

**Authors:** Shui-Wang Qing, Xiao-Ping Ju, Yang-Sen Cao, Huo-Jun Zhang

**Affiliations:** Department of Radiation Oncology, Changhai Hospital, No.168 Changhai road, Shanghai, China

**Keywords:** Locally advanced pancreatic cancer, SBRT study protocol

## Abstract

**Background:**

Dose escalation of SBRT for locally advanced pancreatic cancer patients had been reported in several studies in one or three fractions, and phase I protocol was developed to investigate the maximum tolerated dose with CyberKnife for locally advanced unresectable pancreatic cancer patients in five fractions.

**Methods:**

The study is designed as a mono-center phase I study. The primary endpoint is to determine the maximum tolerated dose by frequency of III/IV GI (gastrointestinal) toxicity. Adverse events (AE) according to Common Toxicity Criteria (CTC) version 4. Doses of 7 Gy, 7.5 Gy, 8 Gy, 8.5 Gy, 9 Gy, 9.5Gy x 5 respectively would be delivered while meeting with normal tissue constraints. A minimum of three patients will be included for each dosage level. And an interval is 4 weeks from the first patient treatment to the next patient treatment at each dose level. The maximal tolerated dose will be defined as the dose for which at least two patients in three, or at least three patients in nine, will present with a limiting toxicity.

**Discussion:**

Since the dose and fractions of SBRT treatment for locally advanced pancreatic cancer patients are still unknown, we propose to conduct a Phase I study determining the maximum tolerated dose of CyberKnife SBRT for the treatment of locally advanced pancreatic tumor based on a 5 fractions treatment regimen. This trial protocol has been approved by the Ethics committee of Changhai hospital. The ethics number is 2016-030-01.

**Trial registration:**

Clinical trials number: NCT02716207.

Date of registration: 20 March 2016.

## Background

Pancreatic cancer is the fourth-leading cause of cancer-related death in the world. It is characterized by metastatic spread and local failure and seldom detected in its earlier stages. For locally advanced stage pancreatic cancer, the complete surgical removal is hard to achieve.

Stereotactic body radiotherapy (SBRT) with CyberKnife for locally advanced unresectable pancreatic cancer is a relatively new treatment option made available because of significant improvements in diagnostic imaging and radiation delivery techniques. Different from the conventional radiotherapy, radiation dose is delivered in fewer fractions and higher fractional doses in SBRT. Gurka [1] reported that 14 patients received SBRT with prescription dose of 25 Gy in five fractions with BED (biologically equivalent doses) of α/β = 10 in correspondence to 37.5Gy. Grade 1 to 2 gastrointestinal toxicity (no grade 3 or 4 radiation-related toxicities) was observed 2 weeks after treatment. Two patients had a partial response, and 12 patients were with stable diseases. In the previous dose escalation study, a single fraction upto15 Gy, 20 Gy, 25 Gywhich is an equivalent BED_10_ to 37.5Gy,60Gy,87.5Gy respectively is recommended by Koong AC [2] and his team. Even though the local control rate is 100%, the follow up is short and the sample size of 15 patients is relatively small. Moreover, the late toxicity is not investigated. And with single fraction scheme, higher late GI toxicities were reported [2–4]. In the meanwhile, investigators [5, 6] from South Korea examined that a Dmax of 35Gy and 38Gy in 3 fractions (BED_10_ to75.8Gy and 86.1Gy) of SBRT correlated with a 5 and 10% rate of grade 3 of gastroduodenal toxicity for abdominal malignant tumor, respectively.

Chuong [7] used 5 fractions to potentially decrease the risk of late normal tissue injury compared with 1 to 3 fractions commonly used in other institutions. Assuming α/β = 3, the EQD2 = nd*(d + α/β/2 + α/β) delivered to normal tissue in this study (using a mean 36.4 Gy in 5 fractions to the high dose PTV) was 75Gy, which is lower than the mean EQD2 from other series, the corresponding values from Boston and Stanford were 92.2Gy (mean, 32.96 Gy in 3 fractions) and 140Gy (mean, 25Gy in 1 fraction), respectively [8, 9]. And a relatively lower incidence of grade ≥3 late adverse effects (5.3% VS 9%) was observed.

## Methods and design

### Study design

The study is designed as a mono-center phase I study to evaluate the maximum tolerated dose for locally advanced unresectable pancreatic cancer treated with CyberKnife SBRT.

### Primary endpoint

To determine the maximum tolerated dose.

### Secondary endpoints

1. To assess the pain intensity by NRS scores.
2. To assess acute and late toxicities following CyberKnife SBRT.
3. Response rate by RECIST criteria.
4. Relapse-free survival (RFS) and overall survival (OS).

### Inclusion criteria

Locally advanced unresectable pancreatic adenocarcinoma which is proved by biopsy.

A life expectancy of >3 months

KPS ≥70

Tumor size <5 cm

Tumor location: head of pancreas

Age of 18–75 years old

Patients must be able to undergo contrast enhanced CT for planning

Absolute neutrophil count ≥ 1,5 × 10^9^cells/L

Leukocyte count ≥ 3.5 × 10^9^cells/L

Platelets ≥ 70 × 10^9^cells/L

Hemoglobin ≥ 8.0 g/dl

Albumin >2.5 g/dL

Total bilirubin < 3 mg/dL

Creatinine < 2.0 mg/dL

INR < 2 (0.9–1.1)

AST < 2.5 × ULN (Upper Limit of Normal) (0–64U/L)

ALT < 2.5 × ULN (0–64U/L).

Both men and women and members of all races and ethnic groups are eligible for this study.

Ability of the research subject or authorized legal representative to understand and the willingness to sign a written informed consent document.

Tumor markers and lab test should be done less than 1 week before recruitment.

### Exclusion criteria

Prior surgery, chemotherapy or radiation for pancreatic tumor.

Prior radiotherapy of the upper abdomen, evidence of metastatic disease such as nodal or distant metastases by abdomen CT and chest CT.

Contraindication to receiving radiotherapy.

Distance between GTV (lesion) and luminal structures (including liver, stomach, duodenum, small or large bowel) is less than 5 mm.

Women who are pregnant or participation in another clinical treatment trial while on study.

Patients in whom fiducial implantation were not possible.

### Radiation treatment planning

SBRT will be delivered on CyberKnife with Synchrony Respiratory Tracking system. The tumor will be tracked with implanted fiducial markers by Fiducial Tracking System. Treatment will be delivered in 5 fractions within 1 to 2 weeks at the discretion of the investigator.

A body fixation (vacuum-bag) will be used in immobilizing the body, the arms (both arms are along the body) and the legs.

Dose will be administered according to the following recommended schedule: Doses of 7 Gy, 7.5 Gy, 8 Gy, 8.5 Gy, 9 Gy, 9.5Gy x 5 with BED_10_ in correspondence to 59.5 Gy, 65.6 Gy, 72 Gy, 78.6 Gy, 85.5 Gy, 92.6 Gy respectively would be delivered while meeting with normal tissue constraints (Table 1). A minimum of three patients will be included for each dosage level. And an interval is 4 weeks from the first patient treatment to the next patient treatment at each dose level. In case patient presents III/IV GI toxicity, three additional patients will be included at the same dose level.

**Table 1:** Diagram of dose escalation

Level	Dose (Gy)	Fractions	Total (Gy)
1	7	5	35
2	7.5	5	37.5
3	8	5	40
4	8.5	5	42.5
5	9	5	45
6	9.5	5	47.5

The maximal tolerated dose will be defined as the dose for which at least patients in 3, or at least three patients in 9, will present with a limiting toxicity.

Margins: The Gross Tumor Volume (GTV) is defined as the visible tumor based on contrast enhanced CT acquired on portal-venous phase. The clinical target volume (CTV) equals the GTV. The planning target volume (PTV) was usually defined as the region of 2–5 mm outside of CTV. When tumor is adjacent to critical organs especially duodenum, we choose to avoid expanding PTV outside of CTV in this direction.

An individualized treatment plan will be developed based on tumor geometry and location. At least 90% volume of PTV should be covered by the prescription dose. The prescription isodose line was limited to 70–75% which will restrict tumor Dmax. e. If dose level violates the constraint of SBRT, the patient will be considered as ineligible for this trial (Table 2).

**Table 2:** Critical structures and constraints

Organ	Dose limits (5 fractions)	Volume
Liver	17.5 Gy	≥700 cc spared
Kidney	<12 Gy	Dmean
Spinal cord	23 Gy	0.35 cc
Spinal cord	27Gy	Dmax
Duodenum	18Gy	5 cc
Duodenum	12Gy	10 cc
Small bowel	19.5Gy	5 cc
Stomach	18Gy	10 cc
Esophagus	19.5Gy	5 cc
Large Bowel	25Gy	20 cc

Based on The Report of AAPM (American Association of Physicists in Medicine) Task Group 101

Fiducial (Soft tissue gold markers, 0.9 × 3 mm, CIVCO, Orange City, lowa 51041 USA) implantation will be done under endoscopic ultrasonography (EUS) guidance. The recommended number of implanted fiducials is 3 (at least 1) which is preferable to be close to, but not in the tumor. A time-period of 4–7 days between implantation and treatment planning CT-scan is recommended.

### Intervention and mode of delivery

The planning CT scan and enhanced pancreatic parenchymal phase CT should be acquired under breath hold (preferably end-expiratory). The scan range includes the whole pancreas, at least 10 cm above and below the tumor. The spiral thin-slice CT was with 1.5 mm slice collimation and images were reconstructed in slices of 1.5 mm at the most. Co-registration of planning CT to contrast enhanced CT is based on fiducial and anatomical (spinal) fusion. With fiducials >3, 3D data could be tracked whereas it is hard to implant three fiducials in clinical practice. If fiducials <3, method of 1 fiducial plus X-sight spine and Synchrony Tracking technique will be applied. Before the treatment, DRR images on spine will be applied to detect the 6-D error and correction will be done thereafter for the X-sight spine tracking on patients’ positioning. During treatment, fiducial tracking will be applied.

### The irradiation planning and radiation techniques

Collimator selection: there are 12 size options for collimators selections from 5 to 60 mm, and it depends on tumor size and the distance to organ at risk (OAR). To improve the treatment efficiency, the collimator size for each enrolled patient is fixed in our study.

MU limit is set up to avoid large MU for single beam. And the total MU is 90,000 for 5 fractions. Maximum MU/beam is limited to 500 and maximum MU/node is limited to 1600.

PTV is taken as the source, and shells are expanded isotropically to constrain high dose volume outside target and increase conformity index of PTV.

OCO (optimize coverage) technique is applied to optimize coverage and conformity.

Beam reduction technique is applied to increase treatment efficiency by deleting beams with small MU.

High resolution is applied to the treatment planning to further optimize and normalize the prescription dose.

### Duration of intervention and evaluation

The duration of treatment will be 1 to 2 weeks. It is delivered every day and 5 times a week. In case of machine breakdown, it will be extended to 2 weeks. The follow-up period will be for 1 year following completion of therapy. The trial is planned to begin on September 2016. There cruitment period of the entire study (27 patients) will take approximately 1 year. Acute and late toxicity according to CTCAE 4.0will be evaluated. Triple phase enhanced abdomen CT scan (<4 weeks), physical examination and lab test will be assessed at 1, 3, 6, 9, 12 months after completion of treatment.

### A quality assurance

SBRT QA group will be established by two radiation oncologists and two physicists.

## Discussion

Since the treatment modality and dose are still under exploratory stage, we propose to conduct a Phase I study determining the maximum tolerated dose of CyberKnife SBRT for the treatment of locally advanced pancreatic tumor based on a 5 fractions treatment regimen. A prescription dose of 35–47.5 Gy in five fractions was chosen, with an equivalent to the traditional dose of 2 Gy in 25–39 fractions of BED_10_. And this is assumed to be the safe and effective dose for unresectable pancreatic cancer patients.

Initial dose: No severe gastrointestinal toxicities were reported when Dmax to the gastrointestinal organs was within 30 Gy [10, 11]. Based on these results, Kavanagh [12] suggested that Dmax of the intestine should be less than 30 Gy in three fractions (BED_10_ to 60 Gy). The initial dose in our study will be defined as 35 Gy in 5 fractions (BED_10_ to 59.5 Gy), which is an equivalent to the traditional dose of 2 Gy in 25 fractions in BED_10_.

The classical “4R” radiobiological factors and linear-quadratic (LQ) model are useful for calculating iso-effect doses in conventional 2Gy fractionated irradiation, which is not enough for estimating hypo-fractionated SBRT [13]. The SBRT may prolong the time of repairing sub-lethal radiation damage compared to conventional irradiation in mamalian cells [14]. Moreover many evidences show that DNA double-strand breaks alone cannot explain the highly effective SBRT [15, 16], which encompasses not only direct but also indirect cell deaths [17] including differential endothelial, overcoming hypoxic radio resistance and activation of immunological pathways.

These biological properties may contribute to reduce normal tissue toxicity and hypofractionation is more effective than conventional radiation therapy.

## Abbreviations

4R: Reoxygenation, repair, repopulation, and redistribution
AAPM: American association of physicists in medicine
BED: Biologically equivalent doses
CT: Computer tomography
CTV: Clinical target volume
EUS: Endoscopic ultrasonography
GI: Gastrointestinal
GTV: Gross tumor volume
INR: International standard ratio
KPS: Karnofsky performance status
LA: Locally advanced
LQ: Linear-quadratic
NRS: Numeric rating scales
OAR: Organ at risk
OCO: Optimize coverage
PTV: Planning target volume
SBRT: Stereotactic body radiotherapy
